# Multi-Objective Optimization of Flow Channels for Uniform and Non-Uniform Heat Sources

**DOI:** 10.3390/mi17080950

**Published:** 2026-08-10

**Authors:** Huxiang Xia, Jian Zhang, Mengyun He, Kaiyuan Du, Jiashu Guo, Ruiling Zhang, Peng Wu, Yue Cao, Zhongjun Yu

**Affiliations:** 1 Aerospace Information Research Institute, Chinese Academy of Sciences, Beijing 100190, Chinahemy@aircas.ac.cn (M.H.); wupeng@aircas.ac.cn (P.W.); yuecao0927@hotmail.com (Y.C.); yuzj@aircas.ac.cn (Z.Y.); 2 School of Electronic, Electrical and Communication Engineering, University of Chinese Academy of Sciences, Beijing 101408, China; zhangjian231@mails.ucas.ac.cn (J.Z.); dukaiyuan18@mails.ucas.ac.cn (K.D.); guojiashu23@mails.ucas.ac.cn (J.G.)

**Keywords:** liquid cooling plate, heat sink, thermal management, topology optimization, multi-objective designs

## Abstract

For high-performance chips with escalating power densities and localized hot spots, liquid-cooled microchannel heat sinks face a critical trade-off between thermal performance and flow resistance. This paper presents a multi-objective topology optimization framework that minimizes average temperature and fluid dissipation while incorporating a high-temperature penalty to suppress extreme local overheating. The model is evaluated under uniform heating and a realistic non-uniform source (AMD EPYC 9965 MCM Chiplet CPU). Results show that the penalty term effectively drives channel evolution toward hot spots, reducing peak temperatures with modest pressure-drop increases. Under uniform heating, optimized channels achieve cooling comparable to serpentine designs but with flow resistance near that of parallel or pin-fin arrays. Under non-uniform heating, the topology adaptively narrows inlet-side channels to redirect coolant to distal CCDs, attaining a peak temperature of 139 °C and a pressure drop of 519 Pa—while conventional parallel and array channels exceed 200 °C due to distal cooling deficiency. This study confirms that topology optimization with localized hot-spot penalties effectively mitigates extreme temperatures, offering a viable path for engineering liquid cold plates in next-generation heterogeneous processors.

## 1. Introduction

The rapid advancement of modern high-performance computing, artificial intelligence processors, and power electronic devices has driven dramatic increases in chip integration and power consumption. As transistor feature sizes continue to shrink and computing capabilities grow exponentially, chip heat flux densities have risen sharply, with localized hot spots becoming an increasingly prominent concern [1]. High heat flux densities not only significantly degrade the performance and operational lifetime of electronic devices but can also trigger reliability issues such as thermal runaway [2]. Consequently, the development of efficient and reliable chip-level thermal management technologies has emerged as a critical bottleneck constraining the next generation of high-performance electronic systems.

Liquid cooling technology, owing to its vastly superior heat dissipation potential compared to air cooling, has become the preferred solution for addressing high heat flux density challenges. Among various liquid cooling approaches, microchannel heat sinks are widely adopted in chip thermal management due to their compact structure, high heat transfer efficiency, and excellent compatibility with chip packaging processes [3,4]. However, as chip heat flux densities continue to escalate and non-uniform heat source distributions grow more complex, conventional straight-channel or serpentine-channel cold plates often suffer from excessively high pressure drops and uneven temperature distributions [5,6]. Serpentine channels, for instance, enhance heat transfer by extending the flow path, but at the cost of significantly increased pressure drops that raise pumping power consumption and leakage risks. Parallel channels, while offering extremely low flow resistance, frequently experience severe heat accumulation in distal regions due to uneven flow distribution, resulting in localized hot spots [7,8].

To overcome the limitations of traditional empirical design approaches, topology optimization (TO) has been introduced into the structural design of liquid cold plates. As a systematic, high-degree-of-freedom inverse design methodology, topology optimization does not presuppose any particular channel configuration. Instead, it determines the optimal distribution of materials (solid and fluid) within a given design domain to achieve target performance metrics—such as minimized thermal resistance or pressure drop—subject to specified physical constraints [9,10].

Since Borrvall and Petersson extended topology optimization to fluid problems [11], the method has been widely applied to various fluid-thermal coupled systems [12,13]. In the field of cold plate design, researchers employing density-based or level-set methods have successfully generated a series of non-intuitive, high-performance branching channel configurations with objectives including minimizing average temperature, minimizing flow resistance, or maximizing heat transfer [14,15].

Zeng et al. verified the efficiency of topology-optimized microchannel heat sinks under forced convection through both experimental and numerical approaches [16]. Furthermore, several studies have begun to address more practical engineering constraints, such as the trade-off between thermal performance and flow resistance in multi-objective optimization [17,18,19,20], as well as performance under non-uniform heat sources [21,22]. For example, Han et al. proposed a fractal-geometry-enhanced topology optimization (FGTO) method that further improves heat transfer performance by explicitly describing the heat exchange area [23].

Despite these notable advances, current topology optimization-based cold plate designs still face several critical challenges. First, regarding the effective suppression of localized hot spots, most multi-objective topology optimization studies focus on the global trade-off between thermal resistance and flow resistance—such as Pareto frontier optimization of global average temperature versus pressure drop—with insufficient attention given to direct control of extreme local temperatures (i.e., hot spots). In actual chip operation, localized hot spots are often the direct cause of device failure [24]. A sole reliance on the average temperature target induces the optimizer to distribute the cooling flow evenly, which may impede the effective mitigation of extreme temperature elevations within specific subdomains. Therefore, effective localized high-temperature suppression strategies must be incorporated into the optimization model.

Second, regarding adaptability from uniform to non-uniform heat sources, existing studies predominantly assume uniform heat flux density to validate algorithmic effectiveness. However, the power dissipation of real high-performance processors—such as CPUs, GPUs, and MCM chips—is highly non-uniform [25,26]. Topology optimization methods must demonstrate their capability to adapt to complex, non-uniform heat source distributions, that is, the ability to autonomously evolve differentiated flow channel layouts in response to the spatial characteristics of the heat sources.

To address these challenges, this paper focuses on liquid cooling for high-power chips and establishes a pseudo three-dimensional multi-objective topology optimization framework incorporating localized hot-spot suppression. Building upon the conventional dual objectives of maximizing global heat transfer and minimizing flow resistance, a high-temperature penalty term is introduced as a third optimization objective to effectively suppress extreme local temperatures within the design domain. The flow channel evolution patterns and thermal performance of this multi-objective model are systematically investigated under both uniform heating and realistic non-uniform heating conditions (using the AMD EPYC 9965 MCM Chiplet CPU as a case study), revealing the mechanisms by which the high-temperature penalty term influences topological structures and temperature distributions. Through systematic comparison with conventional serpentine, parallel, and pin-fin array channels, combined with full three-dimensional conjugate heat transfer simulations, the combined thermal and hydraulic advantages of the designed channels are comprehensively evaluated.

## 2. Topology Optimization Model

### 2.1. Mathematical Model

In topology optimization, the Navier–Stokes equations governs fluid motion, and the continuity equation governs conservation of fluid mass. The fluid is modeled as steady-state, incompressible, and viscous, with a damping term incorporated into the momentum equation to account for flow resistance in the porous medium. The governing equation is expressed as:

(1) $$\rho \left(\frac{\partial u}{\partial t}+u\cdot \nabla u\right)=-\nabla P+\mu \nabla^{2}u-\alpha u$$

(2) ∇·u=0

where ρ is the fluid density, u is the velocity vector, t is time, and μ is the dynamic viscosity. The term α denotes the damping coefficient, representing the inverse permeability of the porous medium.

The material distribution within the domain is determined using the variable density method [27]. The RAMP (Rational Approximation of Material Properties) scheme is employed to interpolate the inverse permeability coefficient:

(3) $$\alpha =\alpha_{min}+\left(\alpha_{max}-\alpha_{min}\right)\frac{q\left(1-\theta \right)}{q+\theta }$$

where the material phase is treated as a porous medium, with the material density represented by the variable θ (0<θ<1). θ=0 indicates a fully solid phase, while θ=1 indicates a fully fluid phase. $\alpha_{min}$ and $\alpha_{max}$ are the minimum and maximum values of α, and q is the penalty parameter.

(4) $$\alpha_{max}=\frac{\mu }{Da\cdot L^{2}}$$

Here, Da is the Darcy number, taken as $10^{-4}$ in this study, and L is the characteristic length of the inlet section.

Physical properties are interpolated using the SIMP method [28]. Material properties for intermediate values of θ are obtained via linear interpolation:

(5) $$k=k_{s}+\theta \left(k_{f}-k_{s}\right)$$

(6) $$C_{p}=C_{ps}+\theta \left(C_{pf}-C_{ps}\right)$$

where k is the thermal conductivity for intermediate θ, $k_{s}$ is the solid-phase thermal conductivity, and $k_{f}$ is the liquid-phase thermal conductivity. $C_{p}$ is the specific heat for intermediate θ, with $C_{ps}$ and $C_{pf}$ denoting the solid-phase and liquid-phase specific heats, respectively.

The energy conservation equation and the interface heat flux continuity equation govern energy conservation and solid–liquid interface temperature transfer.

(7) $$\rho C_{p}\left(\frac{\partial T}{\partial t}+u\cdot \nabla T\right)=\nabla \cdot \;(k\nabla T)\;$$

(8) $$T_{f}={T_{s},\;\;k}_{f}\frac{\partial T_{f}}{\partial n}=k_{s}\frac{\partial T_{s}}{\partial n},\;\mathrm{on}\;\mathrm{solid}–\mathrm{fluid}\;\mathrm{interface}$$

where $T_{f}$ and $T_{s}$ are the temperatures of fluid and solid on solid–fluid interface. n is the unit vector in the normal direction of the interface.

Density filtering and projection are also crucial in topology optimization. This study employs Heaviside projection to process the element-wise θ values [28]:

(9) $$\theta =\frac{tanh\left(\beta \left(\theta_{f}-\theta_{\beta }\right)\right)+tanh\left(\beta \theta_{\beta }\right)}{tanh\left(\beta \left(1-\theta_{\beta }\right)\right)+tanh\left(\beta \theta_{\beta }\right)}$$

where β is the projection parameter controlling the projection sharpness, and $\theta_{\beta }$ is the projection threshold. Values of $\theta_{f}$ above $\theta_{\beta }$ are projected toward 1, while those below are projected toward 0.

In the field of topology optimization, the objectives associated with the optimization simulation encompass two key aspects: minimization of fluid dissipation energy and minimization of average temperature. Fluid dissipation energy represents the kinetic energy dissipated as the fluid moves. In addition to these two objectives, this study incorporates a high-temperature penalty term to prevent localized overheating.

(10) $${obj}_{1}=\int_{\Omega }Td\Omega$$

(11) $${obj}_{2}=\int_{\Omega }\left(\frac{1}{2}\mu \left(\nabla u+\nabla u^{T}\right):\left(\nabla u+\nabla u^{T}\right)+\alpha \left(\theta \right)\left(u\cdot u\right)\right)Td\Omega$$

(12) $${obj}_{3}=\int_{\Omega }{\left(T-T_{\mathrm{pen}}\right)}^{P_{\mathrm{pen}}}*\frac{1}{1+e^{-\left(T-T_{\mathrm{pen}}\right)}}d\Omega$$

where ${obj}_{1}$ is the objective of minimizing average temperature, ${obj}_{2}$ is the objective of minimizing fluid dissipation energy, and ${obj}_{3}$ is the objective of minimizing the high-temperature penalty. $T_{\mathrm{pen}}$ is an engineering threshold. Due to the sigmoid term ($\frac{1}{1+e^{-\left(T-T_{\mathrm{pen}}\right)}}$), the penalty term is activated only when the local temperature exceeds $T_{\mathrm{pen}}$. For those grids whose temperatures do not exceed $T_{\mathrm{pen}}$, their contribution to ${obj}_{3}$ to be zero, thus imposing a penalty term only on localized hot spots. $P_{\mathrm{pen}}$ controls the degree of penalty. In different engineering contexts, $T_{\mathrm{pen}}$ can take different values. For example, in chip heat dissipation, $T_{\mathrm{pen}}$ can be chosen as the maximum allowable temperature of circuits, chips, or solder materials. Here, for the convenience of model comparison, $T_{\mathrm{pen}}$ is set to 75 °C for uniform heat sources and 130 °C for non-uniform.

(13) $${obj}_{total}=a\cdot {obj}_{1}+b\cdot {obj}_{2}+c\cdot {obj}_{3}$$

Here, a, b, and c are objective scaling factors. Since the temperature objective and the flow resistance objective have inherently incompatible units and cannot be directly compared, different scaling factors are assigned to normalize the objectives. The objective weights $\omega_{1}$, $\omega_{2}$, and $\omega_{3}$ represent the proportional relationships among the objective functions and are obtained via the following equations:

(14) $$\omega_{1}=\frac{a\cdot {obj}_{1}}{{obj}_{total}}$$

(15) $$\omega_{2}=\frac{b\cdot {obj}_{2}}{{obj}_{total}}$$

(16) $$\omega_{3}=\frac{c\cdot {obj}_{3}}{{obj}_{total}}$$

Prior to assigning objective weights, the scaling factors must be determined via a pre-computation. Topology optimization is first performed with a=b=c=1, and the three objectives ${obj}_{1}$, ${obj}_{2}$ and ${obj}_{3}$ are monitored until their values stabilize (typically within 10 iterations in this research). The scaling factors are then obtained from $a\cdot {\mathrm{obj}}_{1}=b\cdot {\mathrm{obj}}_{2}=c\cdot {\mathrm{obj}}_{3}$, which corresponds to equal weights ($\omega_{1}=\omega_{2}=\omega_{3}$). For other weight specifications, the corresponding scaling factors can be inversely derived using Equations (14)–(16).

It should be mentioned that waiting for stabilization of the objective values is necessary because during the initial stage of iteration, the material density in the topology optimization domain is uniformly set to its initial value, and no preliminary flow path has yet been formed, which may cause notable numerical fluctuations. These fluctuations primarily arise from the high-temperature penalization objective term. This is because during the early iterations, in the absence of preliminary flow paths, the temperatures at all grids within the integration domain are likely to exceed $T_{\mathrm{pen}}$. Moreover, owing to the exponential enhancement in ${obj}_{3}$, the fluctuations in the objective value can be very large. As a result, in the present research, the objective values at the 10th iteration are used as the basis for computing the scaling factors.

### 2.2. Computational Procedure and Parameters

**Figure 1:**
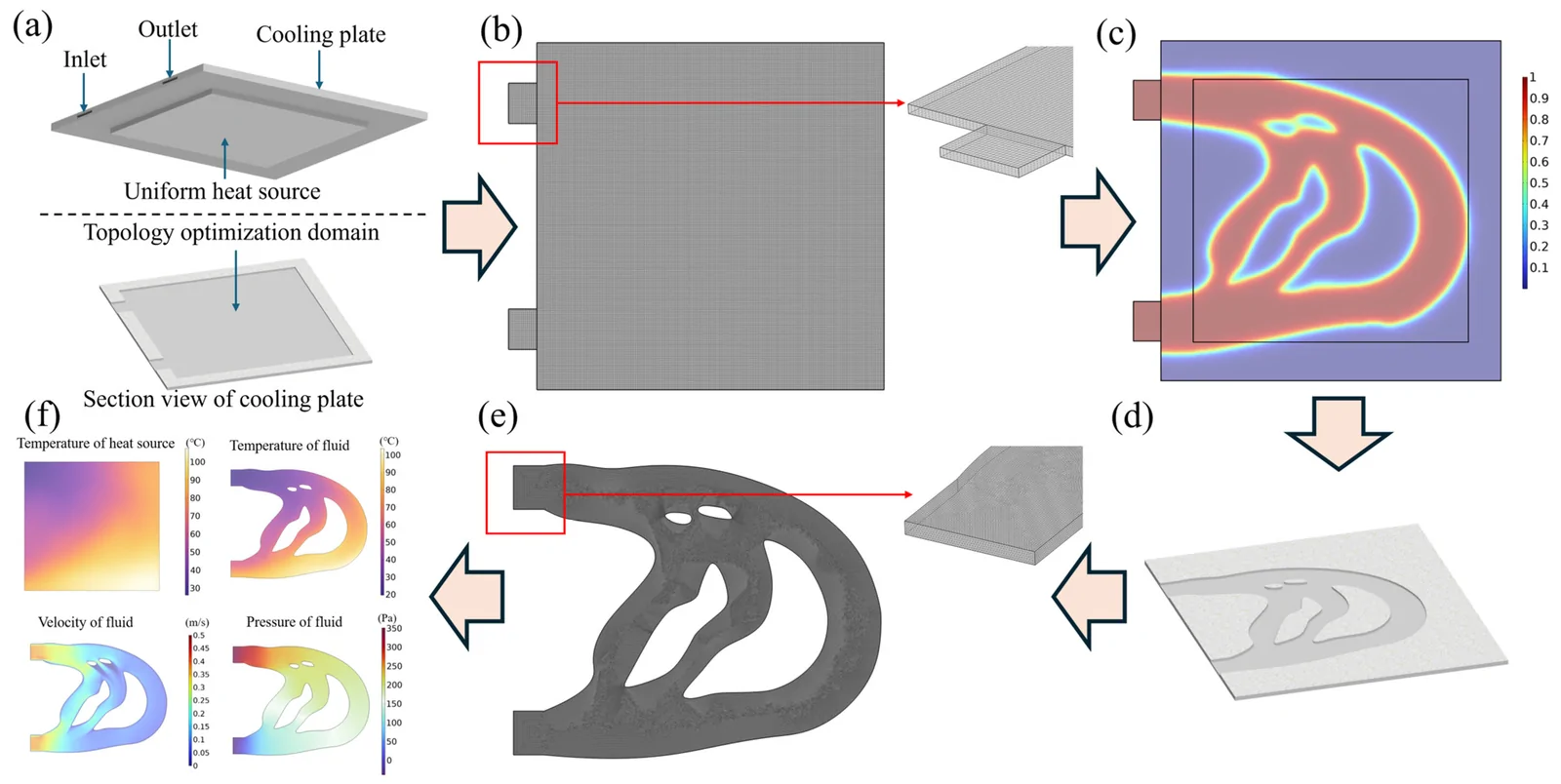
Topology optimization model workflow: (**a**) three-dimensional geometric model; (**b**) topology optimization domain meshing (single-layer mesh); (**c**) topology optimization computation; (**d**) topology optimization mapping mesh; (**e**) topology-optimized channel meshing (fine multi-layer mesh); (**f**) thermal and fluid simulation.

**Figure 2:**
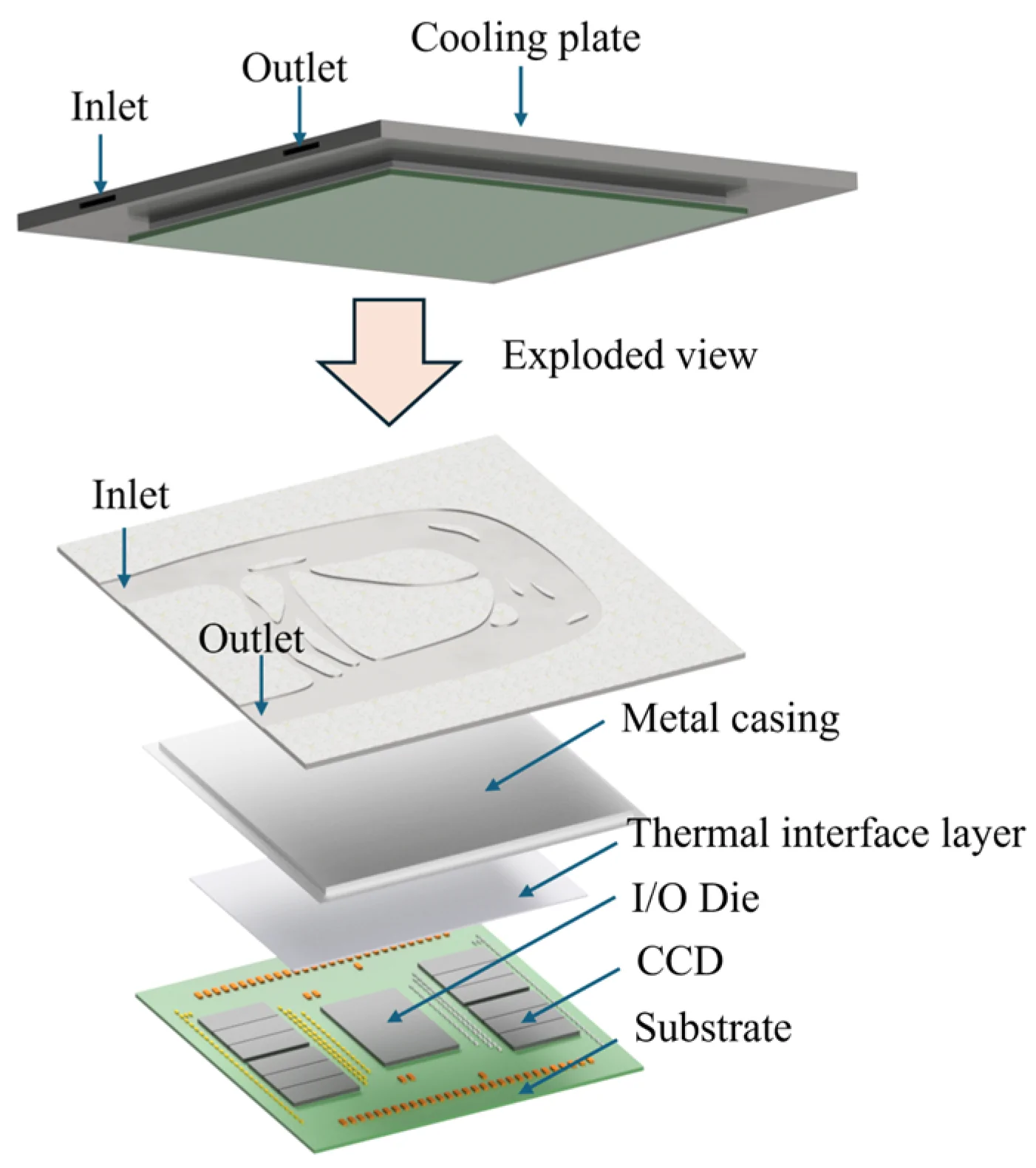
Realistic non-uniform heat source model: AMD EPYC 9965 Multi-Chip Module (MCM) Chiplet.

The design procedure for channel topology optimization is illustrated in Figure 1. The process begins with the establishment of a three-dimensional geometric model. Two heat source configurations are presented in this study: Figure 1a shows the uniform heat source configuration, which facilitates parametric analysis of the topology optimization framework, while Figure 2 presents the realistic non-uniform heat source configuration based on the AMD EPYC 9965 Multi-Chip Module (MCM) Chiplet CPU (Advanced Micro Devices, Inc., US), which integrates 12 Zen 5c CCDs and a central I/O Die.

The total power is 500 W for both the uniform and non-uniform heat sources, with identical envelope dimensions of 72 mm × 75 mm. The topology optimization procedure remains consistent across both cases, as illustrated in Figure 1b–f, with differences arising only in the heat source distribution and internal structural details.

The topology optimization domain is established within the cooling plate, with dedicated meshing strategies applied. In practical fabrication, only a single-layer channel structure is manufactured. Therefore, the mesh within the topology design domain consists of a single layer. The upper and lower walls of the topology domain are set as slip boundaries. During conventional CNC machining, the milling cutter is generally not tilted. Hence, the channel cross-section remains uniform through the thickness direction. Accordingly, we retain only a single layer of elements in that direction, which makes the fluid flow calculation essentially equivalent to that of a two-dimensional model. Nevertheless, we adopt a three-dimensional model primarily to enhance the accuracy of temperature field evaluation, as it can more realistically capture the three-dimensional heat transfer process.

The MMA method and the adjoint method are applied for numerical optimization and sensitivity calculation. The optimization tolerance in this study is set to 10^−9^, and the internal tolerance factor is set to 0.1. Topology optimization typically requires over 150 iterations (approximately 27 h on a CPU i9-14900HX, Intel Corporation, USA, with 32 threads). To reduce computation time, the mesh size should not be excessively large. The topology computation domain is discretized using regular hexahedral elements with edge lengths not exceeding 0.3 mm.

The boundary and source conditions adopted in the present model are summarized in Table 1. The inlet volumetric flow rate is specified as 0.15 L/min. This value is chosen as the evaluation benchmark because, under the current serpentine channel configuration and a uniform heat source, the resulting maximum temperature reaches approximately 73 °C—a level that satisfies the thermal requirements of numerous high-power electronic devices, such as T/R modules [22]. For surfaces exposed to ambient air (e.g., the cold plate), natural convection is assumed, with a convective heat transfer coefficient of 3 W/(m^2^·K). It should be noted, however, that within the typical natural-convection range (2–15 W/(m^2^·K)), the fraction of heat removed by air convection is marginal compared with that carried away by the coolant. Consequently, its effect on chip peak temperature is negligible: the difference in maximum temperature between convective heat transfer coefficient of 2 and 15 W/(m^2^·K) is less than 0.5 °C, which is substantially smaller than the variations induced by different channel geometries. Both the uniform and non-uniform heat source cases (AMD EPYC 9965 processor) are assigned a total power of 500 W [29]. For the EPYC 9965, the I/O Die is estimated to dissipate 80 W, while the remaining ~420 W is attributed to the CCDs. Under actual operating conditions, the power distribution of this processor varies with workload. However, given that this study aims to investigate the thermal dissipation characteristics under different channel configurations, rather than to model the dynamic power behavior of the processor, the detailed heat allocation across various workloads is not considered. The computational parameters used for topology optimization are listed in Table 2, along with the associated material properties. Given that the exact material data for the I/O Die and CCD have not been publicly released, single-crystal silicon properties are employed as a reasonable approximation.

After specifying the boundary and source conditions (Table 1), topology optimization computational parameters (Table 2) and material properties (Table 3) the topology optimization is performed, yielding a flow channel topology similar to that shown in Figure 1c. The detailed geometric information of each component is shown in Table 4. The ambient temperature is 25 °C, and convective heat transfer is applied to all exposed surfaces with a convective heat transfer coefficient of 3 W/(m^2^·K).

The topology is then mapped onto the actual three-dimensional model through projection, producing the channel structure shown in Figure 1d. In the simulation, this channel structure is remeshed with significantly finer resolution than that used in topology optimization. Tetrahedral elements with a maximum element size below 0.02 mm are employed.

According to the Reynolds number formula Re=ρvL/μ and the characteristic length formula L=4A/P (where A is the flow cross-sectional area and P is the wetted perimeter), the Reynolds number at the inlet of the model is calculated to be 454.5, which is in the laminar flow regime. At the local branches of the topology optimization, the maximum average velocity in the flow passage is approximately 0.45 m/s, the channel width is 2.9 mm, and the characteristic length is 1.49 mm, and the Reynolds number is 669.2. Both the inlet and the local branches satisfy the laminar flow requirement. Therefore, the fluid model adopts the laminar flow assumption.

### 2.3. Model Validation

To exclude mesh dependency and ensure the reliability of the numerical results, a mesh independence analysis was performed on the topology optimization model subject to a uniform heat source, with all physical properties and input conditions held constant. The results are summarized in Table 5. The temperature bias and pressure drop bias are defined as the relative differences between the values obtained with a given mesh and those computed using the finest mesh (14.5 million elements). For mesh counts exceeding 7.2 million, both biases fall below 0.1%. In all cases investigated, the minimum number of elements in the channel region is 5.1 million, while the total mesh counts are at least 8.2 million for uniform heat source models and 9.6 million for non-uniform heat source models.

The governing equations of the present pseudo-three-dimensional (pseudo-3D) topology optimization model are established following the bi-objective two-dimensional (2D) formulation proposed by Shi et al. [22]. Building upon their 2D framework, we developed a pseudo-3D version and incorporated an additional objective function penalizing high temperatures. In their original work, Shi et al. conducted experimental validation, and their simulation results showed good agreement with measured temperatures and pressure drops. To validate the accuracy of our model, we reconstructed an identical topology optimization configuration to that of Shi et al., employing exactly the same boundary conditions, material properties, and computational parameters.

**Figure 3:**
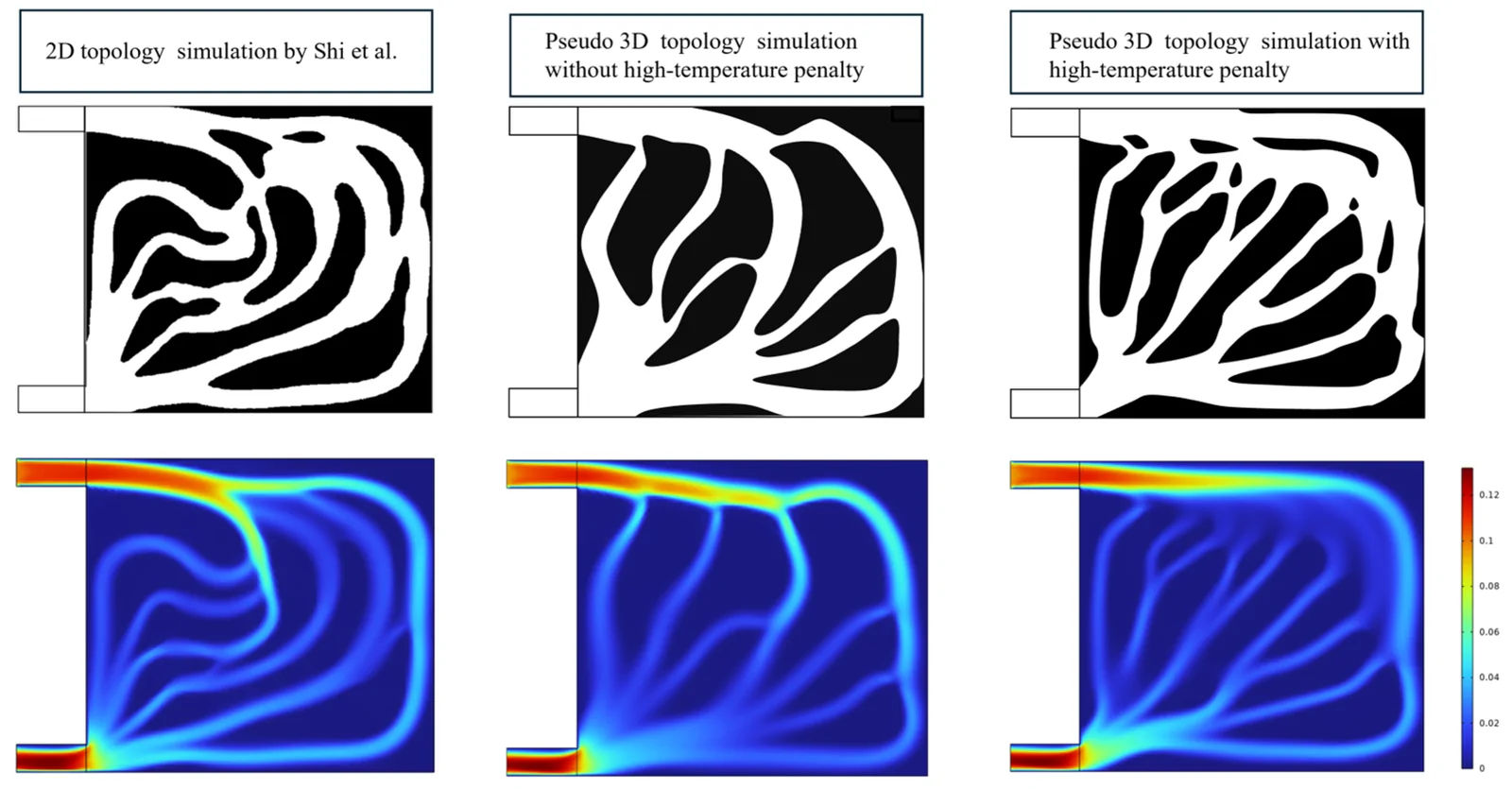
Comparison of topology optimization morphologies [22].

Figure 3 compares the topological patterns obtained from three cases: the 2D simulation of Shi et al., the pseudo-3D simulation without high-temperature penalty, and the pseudo-3D simulation with high-temperature penalty. All three models exhibit uniformly distributed flow channels. It is worth noting that, owing to inherent differences in longitudinal heat transfer between 2D and 3D geometries, the channel distributions are not expected to be perfectly identical even under strictly consistent parameter settings.

**Figure 4:**
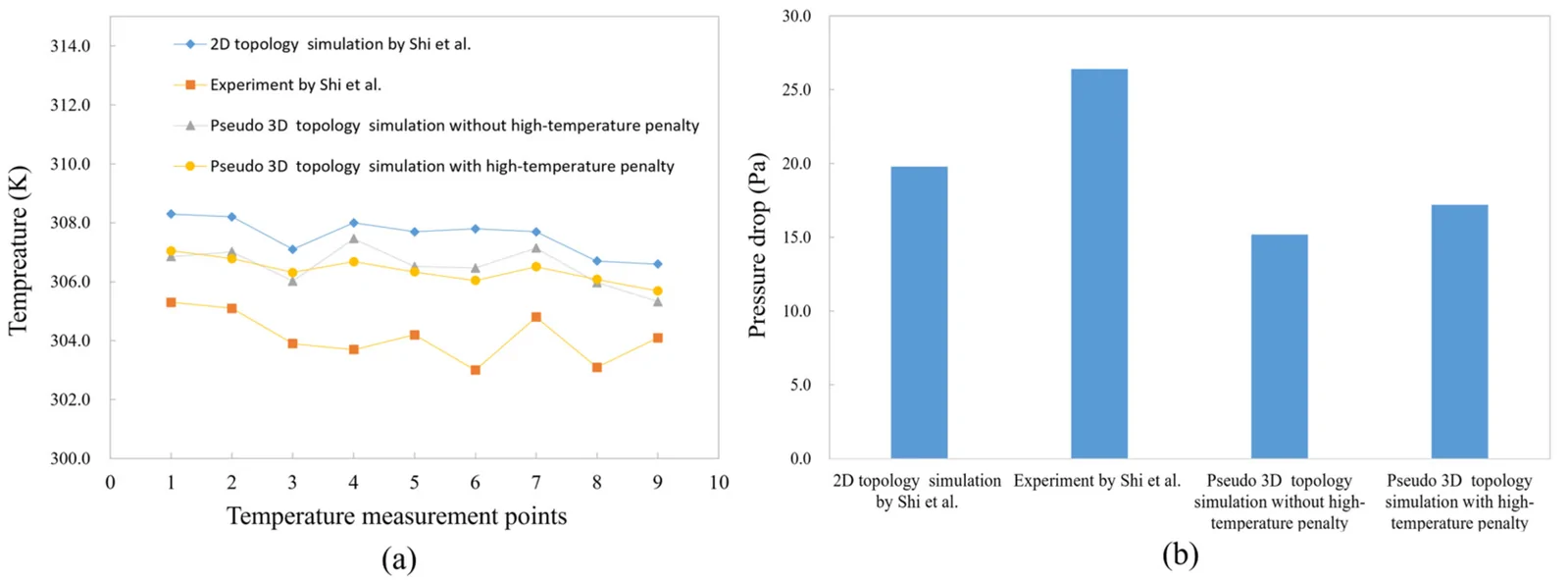
Comparison of topology optimization results: (**a**) Comparison of temperature; (**b**) Comparison of pressure drop [22].

Figure 4 presents the simulated and experimental temperatures at the measurement points, as well as the pressure drops, reported by Shi et al., alongside our pseudo-3D predictions. The results indicate that both pseudo-3D simulations (with and without the high-temperature penalty) yield values lying between the numerical and experimental results of Shi et al. The channel pattern obtained with the high-temperature penalty is notably more dispersed than that without it, leading to improved temperature uniformity, which is also reflected in the temperature distributions at the measurement points shown in Figure 4a. However, the model without the high-temperature penalty produces relatively thicker flow paths and shorter detour distances, resulting in a somewhat lower flow resistance. Specifically, for the pseudo-3D model without the high-temperature penalty, the maximum deviation from the experimental data at any measurement point is 3.5 °C, the maximum deviation from the simulation results of Shi et al. is 1.4 °C, and the pressure-drop deviation is 4.6 Pa. For the pseudo-3D model with the high-temperature penalty, the corresponding maximum deviations are 3.0 °C from experiments, 1.8 °C from the simulations of Shi et al., and 2.6 Pa for pressure drop. Notably, both our simulated pressure drops and those of Shi et al. are slightly lower than their experimental measurements, which can be plausibly attributed to the fact that our numerical model does not account for the pressure losses from connectors and built-in tubing, whereas such losses were inevitably included in the total measured pressure drop during the experiments.

In summary, the mesh independence analysis confirms that the adopted mesh resolutions are sufficient to eliminate discretization-induced errors, and the comparative study against both the 2D model of Shi et al. and their experimental data demonstrates that the present pseudo-3D model yields result in close agreement with established benchmarks. These validations substantiate the accuracy and reliability of the proposed model for the subsequent topology optimization investigations.

## 3. Results and Discussion

### 3.1. Topology Optimization with Uniform Heat Source

The topology optimization framework adopted in this study is based on a multi-objective function that comprehensively accounts for global heat dissipation, flow loss, and localized high-temperature suppression. The objective function is a weighted sum of three terms, with weight coefficients $\omega_{1}$ (thermal dissipation term), $\omega_{2}$ (flow resistance term), and $\omega_{3}$ (high-temperature penalty term). To clarify the influence of each penalty term on the final channel configuration, a parametric study is first conducted under uniform heat source conditions (total power 500 W).

**Figure 5:**
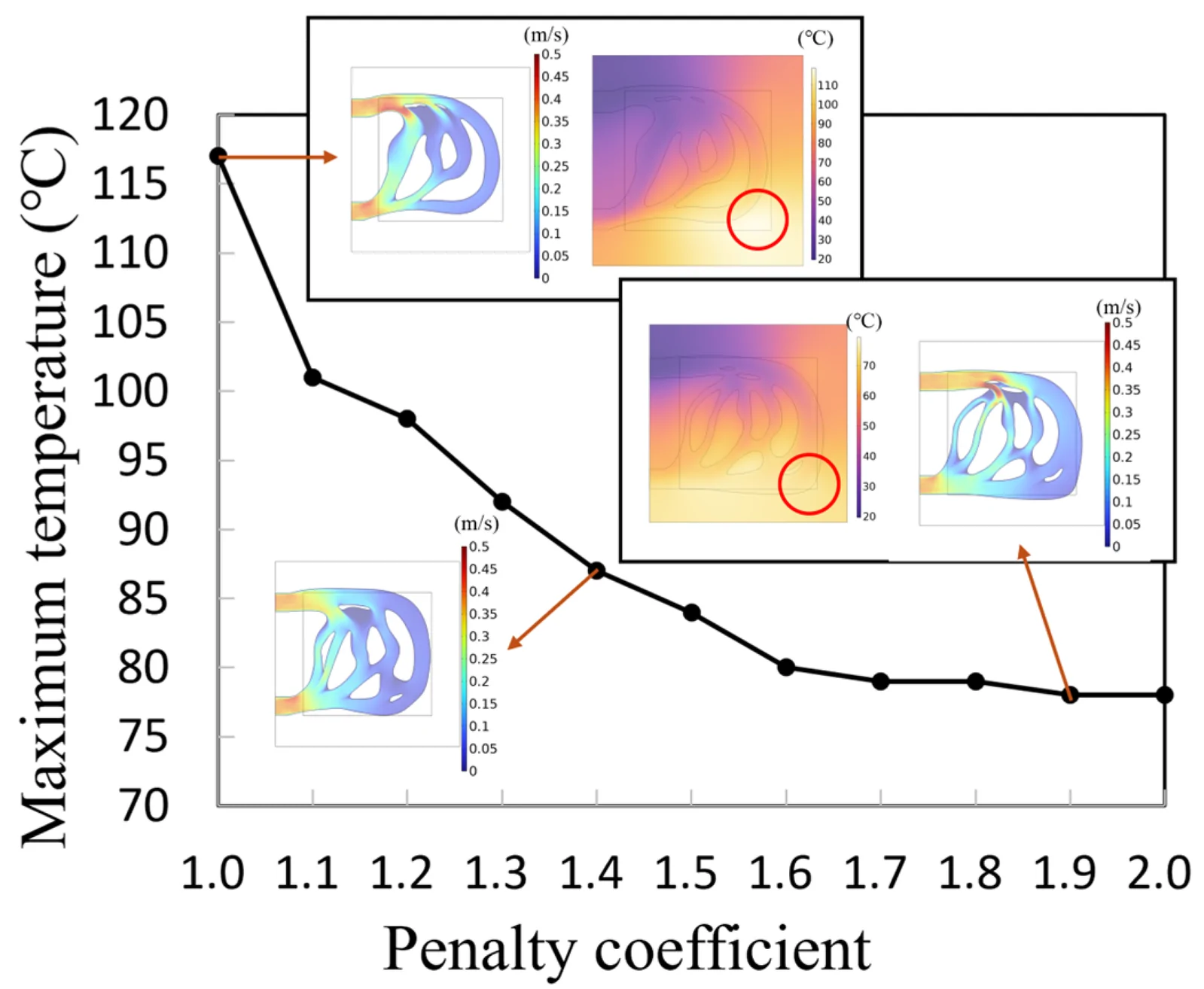
Maximum heat source temperature under different high-temperature penalty exponents.

We first analyze the channel thermal performance under different high-temperature penalty exponents $P_{\mathrm{pen}}$, with $\omega_{1}=0.4$ and $\omega_{2}=0.3$, $\omega_{3}=0.3$ and a penalty threshold $T_{\mathrm{pen}}$ of 75 °C. Figure 5 illustrates the variation in the maximum heat source surface temperature with the high-temperature penalty exponent $P_{\mathrm{pen}}$, which controls the exponential penalty intensity for temperatures exceeding the threshold. When the penalty coefficient $P_{\mathrm{pen}}$ is set to 1, the temperature penalty in ${obj}_{3}$ is not exponentially amplified. The resulting channel exhibits a typical branching network, but the maximum temperature reaches approximately 117 °C. This indicates that without exponential amplification of the temperature penalty, the optimizer exhibits relatively weak sensitivity to localized hot spots, and thus tends to distribute cooling flow uniformly, responding inadequately to extreme local temperature rises.

Once the penalty term $P_{\mathrm{pen}}$ > 1—even with a modest exponent of $P_{\mathrm{pen}}=1.1$—the maximum temperature drops sharply to approximately 101 °C. This abrupt reduction arises from the mathematical construction of the penalty: any nodal temperature exceeding $T_{\mathrm{pen}}$ in the objective function incurs an exponentially increasing penalty, compelling the algorithm to redistribute solid/fluid materials toward regions prone to isolated high-temperature zones (the lower-right region in Figure 5, marked by a red circle). As $P_{\mathrm{pen}}$ continues to increase, the maximum temperature decreases further, though the improvement gradually diminishes and asymptotically approaches the threshold temperature. Topological configuration analysis reveals that the introduction of the penalty term causes the channels to migrate noticeably toward the lower-right corner of the design domain—a region consistently vulnerable to high temperatures even under uniform heating. This observation confirms that the high-temperature penalty term effectively mitigates localized overheating.

**Figure 6:**
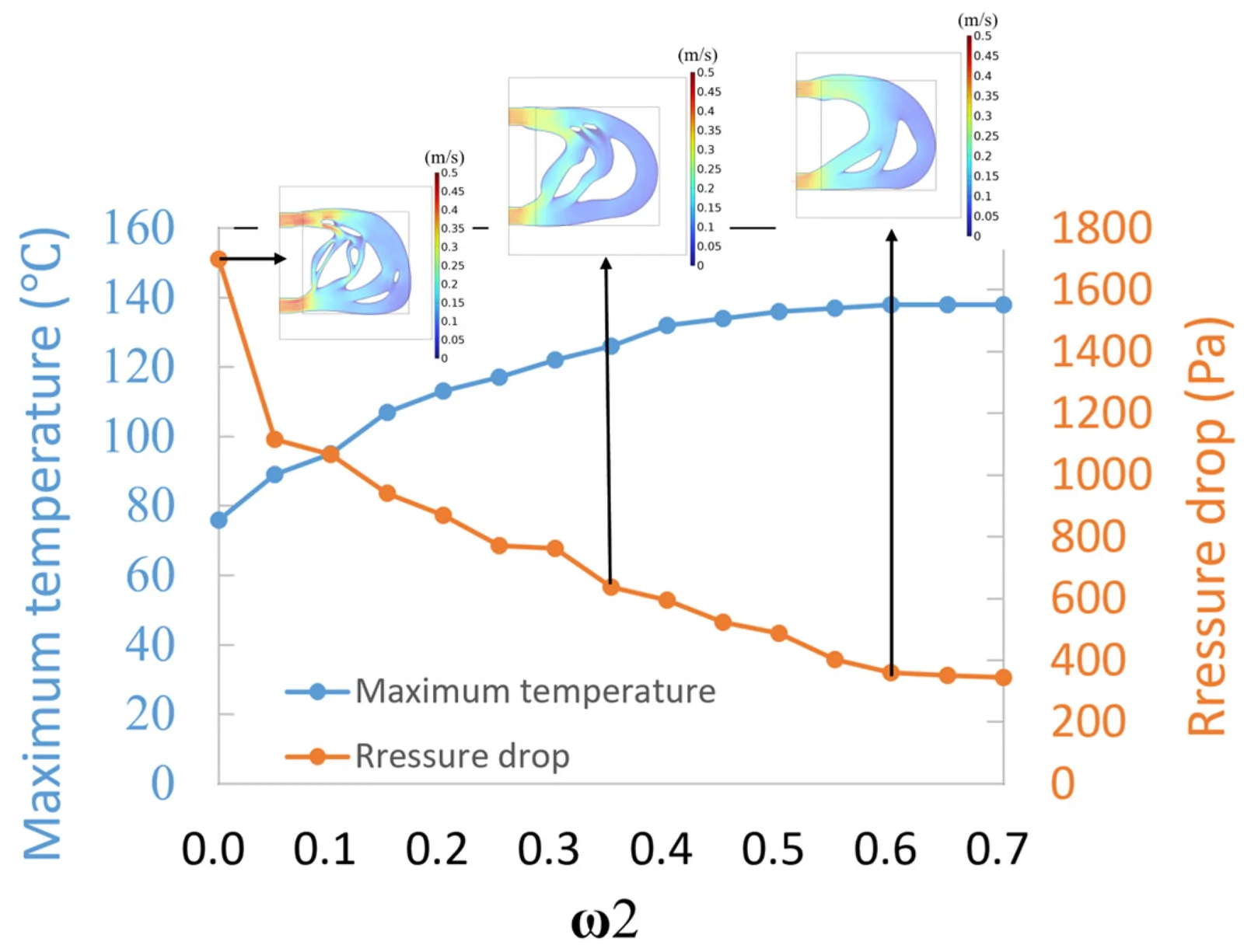
Maximum heat source temperature and pressure drop under different flow resistance optimization coefficients.

Figure 6 presents the relationship between the maximum heat source temperature and the inlet-outlet pressure drop under varying flow resistance weight coefficients. Here, the high-temperature penalty exponent $P_{\mathrm{pen}}$ is fixed at 1.1, and the penalty threshold temperature is set to 65 °C. This threshold is chosen to ensure that the penalty term remains active across all cases, thereby eliminating the interference of penalty deactivation. Meanwhile, $\omega_{3}$ is fixed at 0.1, yielding $\omega_{1}=0.9-\omega_{2}$.

The results indicate that increasing the flow resistance weight systematically reduces the pressure drop. When $\omega_{2}$ dominates, the pressure drop reaches a minimum of approximately 380 Pa. Conversely, when heat dissipation is prioritized exclusively, the maximum temperature can be reduced to approximately 79 °C, but the pressure drop rises to about 1720 Pa.

Although the pressure drop varies by a factor of three, it is noteworthy that even the topology least concerned with flow resistance ($\omega_{2}=0$) exhibits a pressure drop far below 1800 Pa—significantly lower than the values obtained for conventional serpentine channels in Section 3.2. This suggests that topology-optimized channels inherently possess streamlined, sharp-turn-free geometries, which are naturally conducive to low flow resistance. In other words, topology optimization permits aggressive pursuit of thermal performance improvements without incurring prohibitive pumping power penalties.

### 3.2. Evaluation of Channel Configurations Under Uniform Heat Source

**Figure 7:**
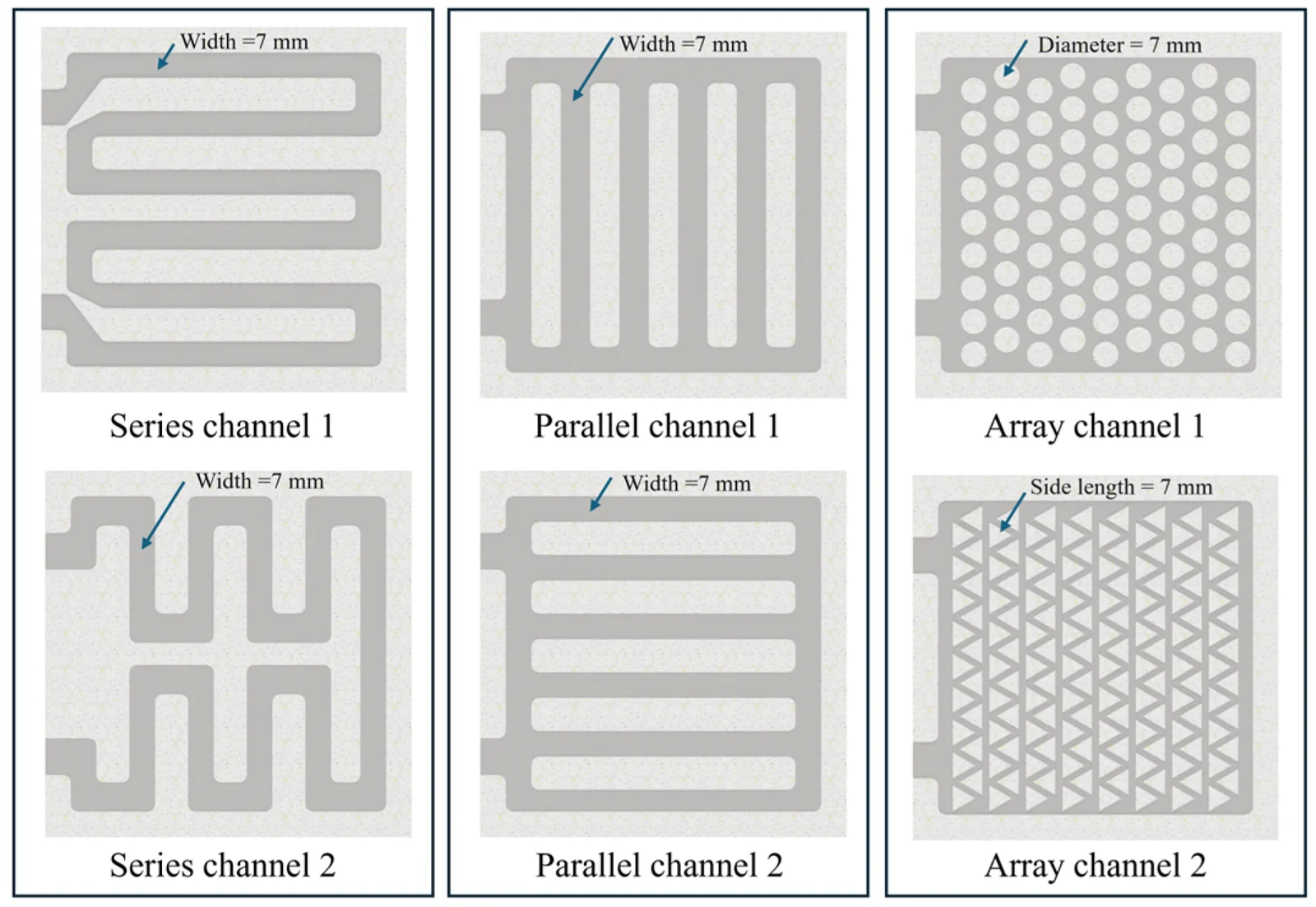
Conventional channels.

**Figure 8:**
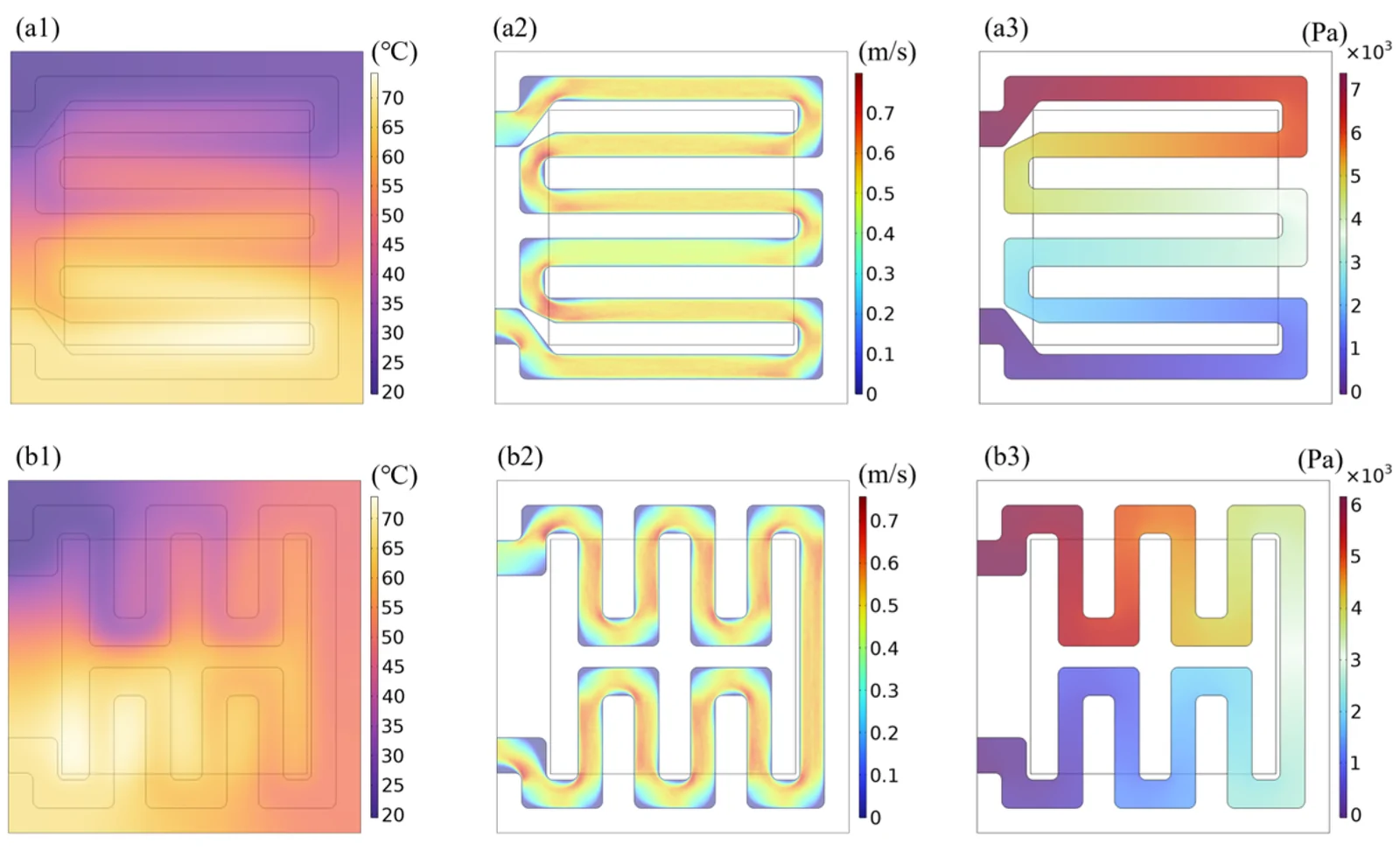
Series channel simulation results: (**a1**) Series channel 1 temperature distribution, (**a2**) velocity distribution, (**a3**) pressure distribution; (**b1**) Series channel 2 temperature distribution, (**b2**) velocity distribution, (**b3**) pressure distribution.

**Figure 9:**
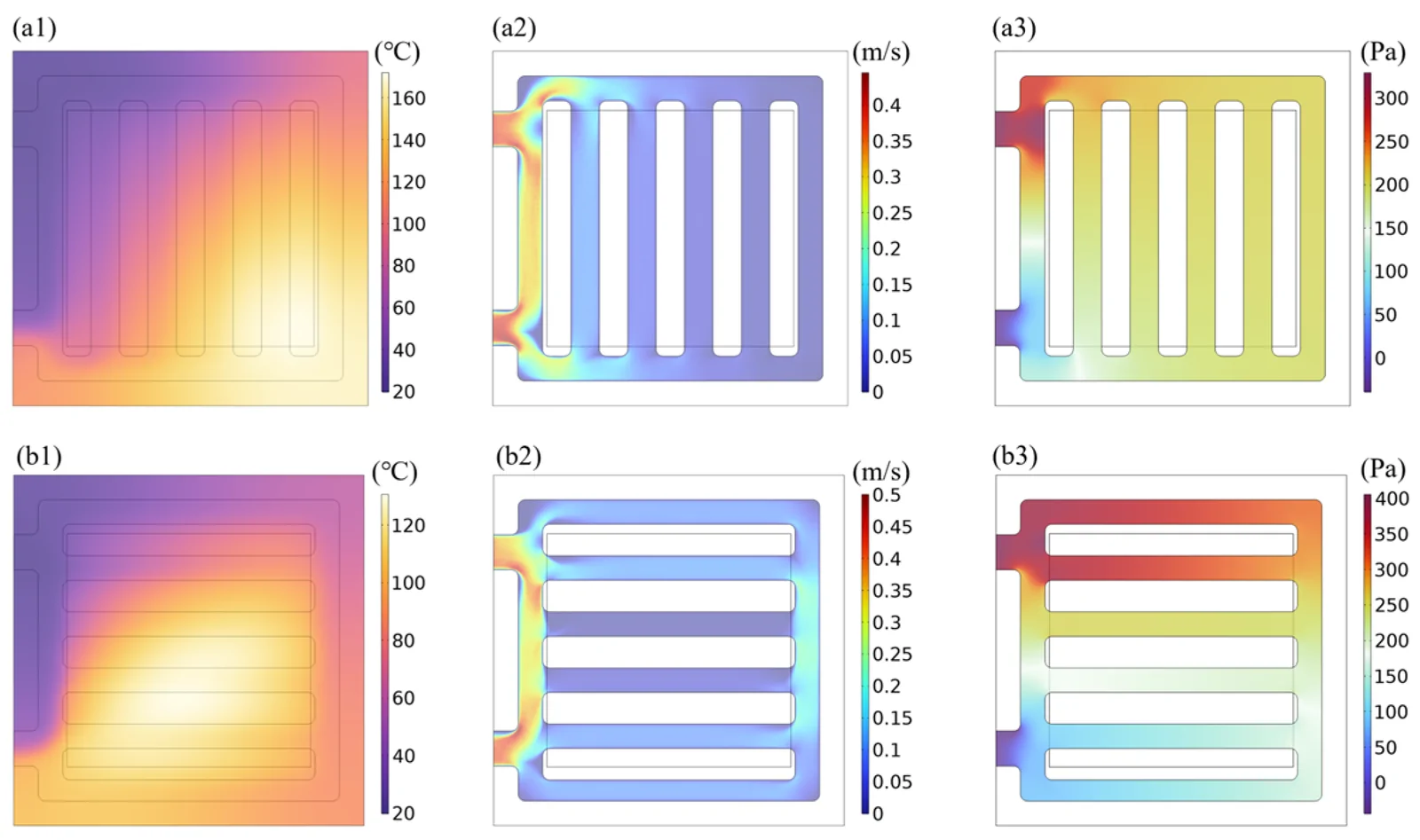
Parallel channel simulation results: (**a1**) Parallel channel 1 temperature distribution, (**a2**) velocity distribution, (**a3**) pressure distribution; (**b1**) Parallel channel 2 temperature distribution, (**b2**) velocity distribution, (**b3**) pressure distribution.

**Figure 10:**
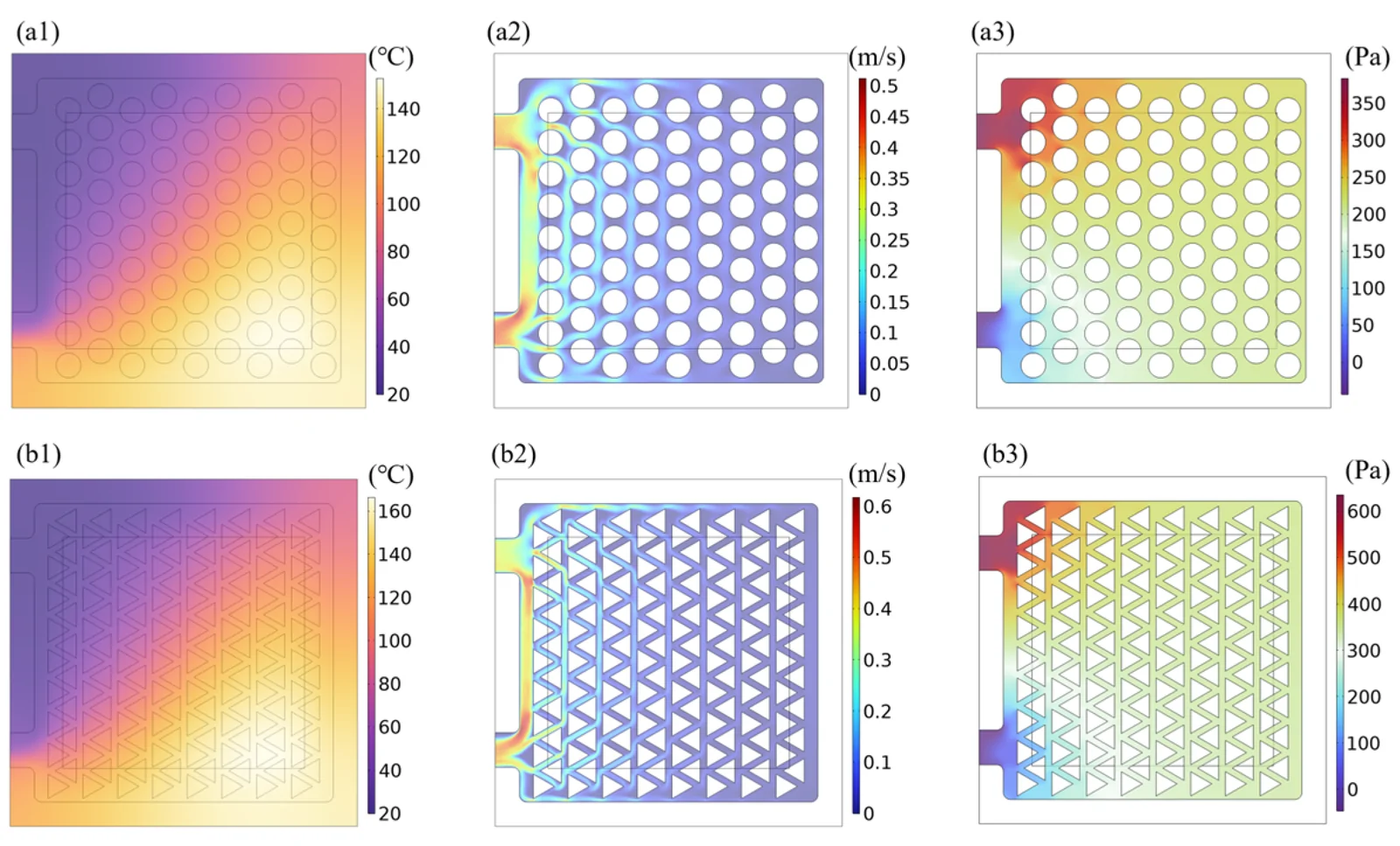
Array channel simulation results: (**a1**) Array channel 1 temperature distribution, (**a2**) velocity distribution, (**a3**) pressure distribution; (**b1**) Array channel 2 temperature distribution, (**b2**) velocity distribution, (**b3**) pressure distribution.

**Figure 11:**
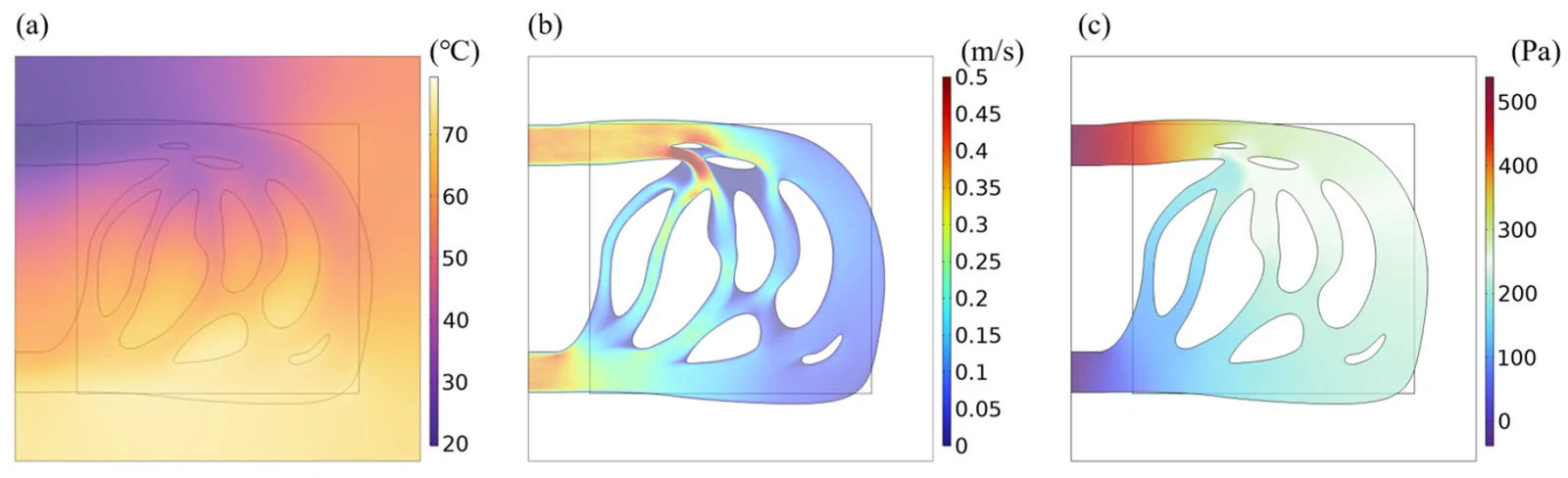
Topology-optimized channel: (**a**) temperature distribution, (**b**) velocity distribution, (**c**) pressure distribution.

To establish a performance baseline and objectively evaluate the topology-optimized channels, six conventional channel configurations are designed, as shown in Figure 7, including serpentine, parallel, and pin-fin array types, all under identical boundary conditions. The area fraction occupied by conventional channels is maintained at approximately 45%, consistent with the fluid fraction constraint within the topology optimization domain, with a channel-to-plate-edge distance of 7 mm. The temperature, velocity, and pressure distributions for these conventional channels are presented in Figure 8, Figure 9 and Figure 10. Figure 11 shows the multi-objective topology optimization result, with $\omega_{1}=0.4$, $\omega_{2}=0.3$, $\omega_{3}=0.3$, a high-temperature penalty exponent $P_{\mathrm{pen}}$ of 1.9, and a high-temperature penalty threshold of 75 °C.

It should be noted that the conventional channel configurations compared in this section were not subjected to full parametric optimization under the same non-uniform heating conditions. The comparison presented here is therefore not intended to demonstrate that the topology-optimized channel outperforms the globally optimal conventional design for this specific heat source distribution. Rather, it serves to illustrate a key advantage of the topology optimization methodology: unlike conventional designs, which typically require heuristic manual adjustments for each new heat source distribution, the topology-optimized channel—driven by the high-temperature penalty term—autonomously adapts its internal flow network to arbitrary spatial power maps without case-specific redesign. This universality represents a fundamental distinction between systematic topology optimization and empirical channel design.

**Figure 12:**
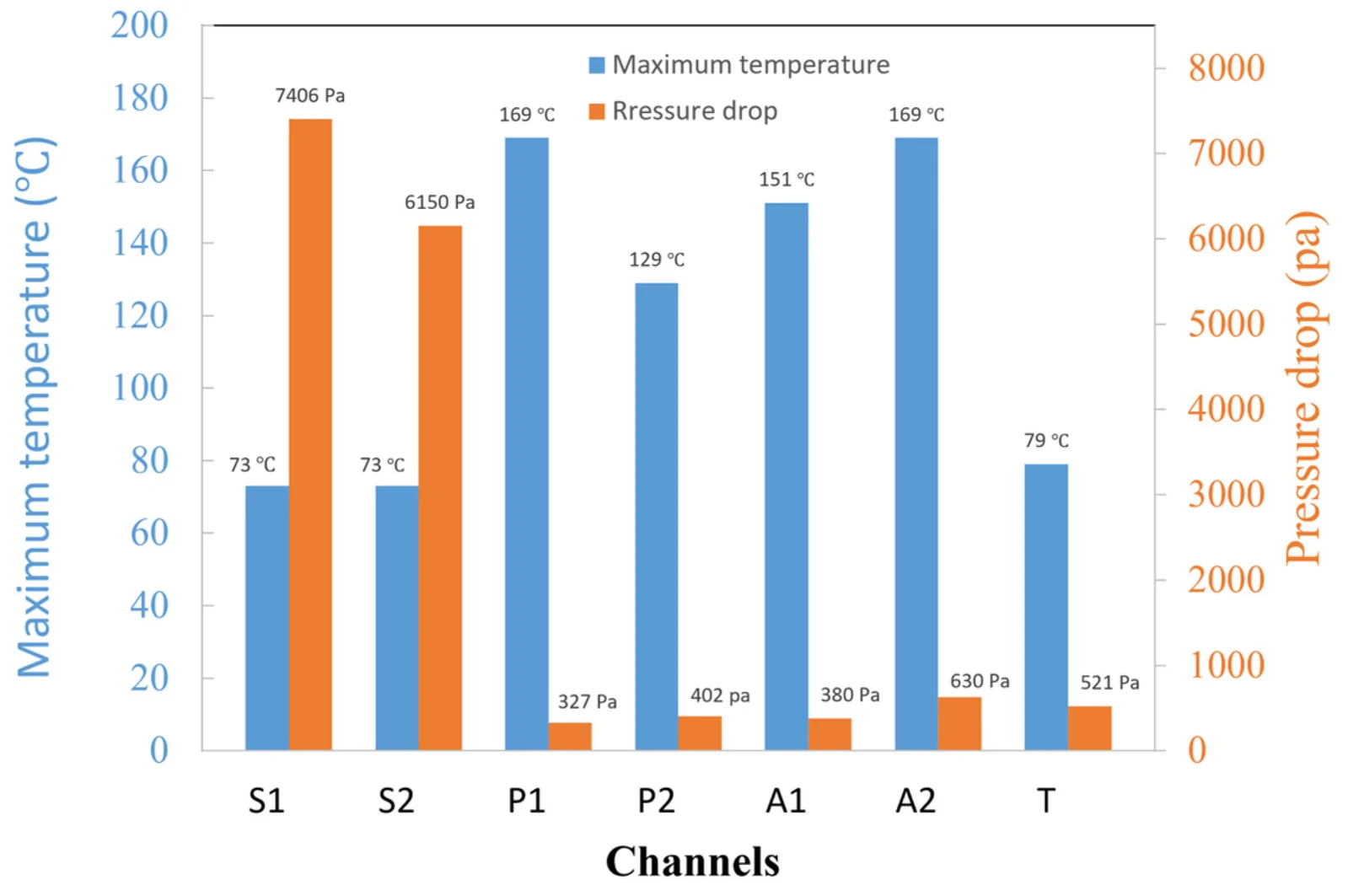
Maximum heat source temperature and pressure drop for different channel configurations under uniform heating.

Figure 12 summarizes the comprehensive performance of each channel in terms of maximum heat source temperature and inlet-outlet pressure differential. Among conventional configurations, the two serpentine channels exhibit the lowest maximum temperatures, both approximately 73 °C. This superior thermal performance is attributed to the prolonged fluid residence time along the serpentine path, during which the coolant traverses the entire heating surface multiple times, thereby enhancing convective heat transfer. However, this benefit comes at a substantial cost: the pressure drops for the serpentine channels reach approximately 7406 Pa and 6150 Pa, respectively—nearly an order of magnitude higher than other channel types. Such high flow resistance imposes stringent requirements on pumping systems and significantly increases leakage risks in large-scale deployments.

The two parallel channels exhibit pressure drops of only 327 Pa and 402 Pa, reflecting extremely low hydraulic impedance. However, their thermal performance deteriorates severely, with maximum temperatures reaching 169 °C and 129 °C, respectively. The fundamental cause lies in the short, straight flow paths from inlet to outlet, which lead to pronounced flow maldistribution—coolant preferentially flows through the low-resistance channels near the inlet, leaving the distal heating regions inadequately cooled—a “short-circuiting” effect exacerbated by the inability of the inlet plenum to enforce uniform flow distribution.

The circular pin-fin array channel yields a maximum heat source temperature of 151 °C with a pressure drop of 380 Pa, while the triangular array performs even worse on both metrics, at 169 °C and 630 Pa. The poor performance of the triangular array is attributed to the sharp corners that induce flow separation and recirculation zones, increasing both the thermal boundary layer thickness and form drag. Although these arrays provide larger heat transfer areas compared to serpentine and parallel channels, their cooling capacity remains limited because the flow never reaches the distal regions of the heat source in sufficient quantity.

The topology-optimized channel achieves a maximum temperature of 79 °C—slightly higher than the serpentine channels—while the pressure drop is only 521 Pa. This places the topology-optimized channel in a unique performance regime: it offers cooling performance approaching that of serpentine channels, yet its hydraulic cost is only marginally higher than that of parallel and circular array channels.

Examining its channel configuration reveals a distinct hierarchical branching structure: the inlet main trunk bifurcates into multiple secondary branches that extend to the distal regions of the design domain (lower-right corner), where the high-temperature penalty term exerts its strongest influence. The channel cross-sections in the distal regions are noticeably widened to compensate for the increased flow resistance along the longer paths, preventing excessive pressure rise. Furthermore, all bifurcations feature smooth transitions without sharp turns, eliminating flow separation and localized losses. These characteristics fully demonstrate the core advantage of topology optimization—its ability to autonomously discover non-intuitive channel layouts that simultaneously enhance cooling along extended paths and maintain streamlined hydraulic performance.

### 3.3. Evaluation of Channel Configurations Under Non-Uniform Heat Source

The uniform heat source results in the previous section primarily serve to validate the fundamental effectiveness of the optimization methodology. However, actual high-performance processors typically exhibit highly non-uniform power distributions. To address this, we extend our investigation to a representative real-world thermal load—the AMD EPYC 9965 Multi-Chip Module (MCM) Chiplet CPU, which integrates 12 Zen 5c CCDs and a central I/O Die. The total power remains 500 W, with the CCDs dissipating 420 W (approximately 35 W per CCD) and the I/O Die dissipating 80 W. Compared to the uniform heating scenario, this configuration poses a more severe thermal challenge, as the heat generation is concentrated in discrete high-heat-flux regions rather than being uniformly distributed.

**Figure 13:**
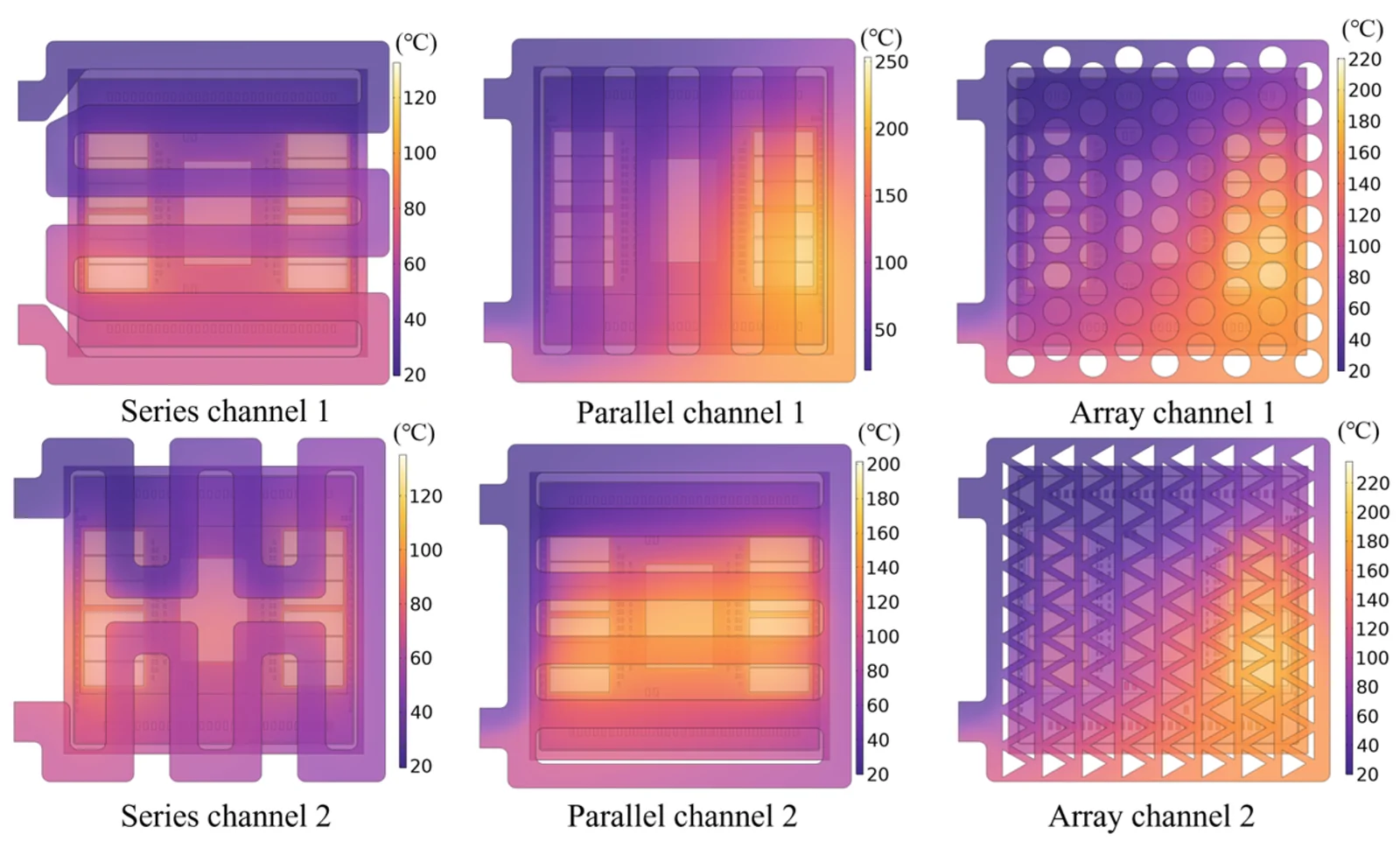
Temperature distributions of conventional channels.

**Figure 14:**
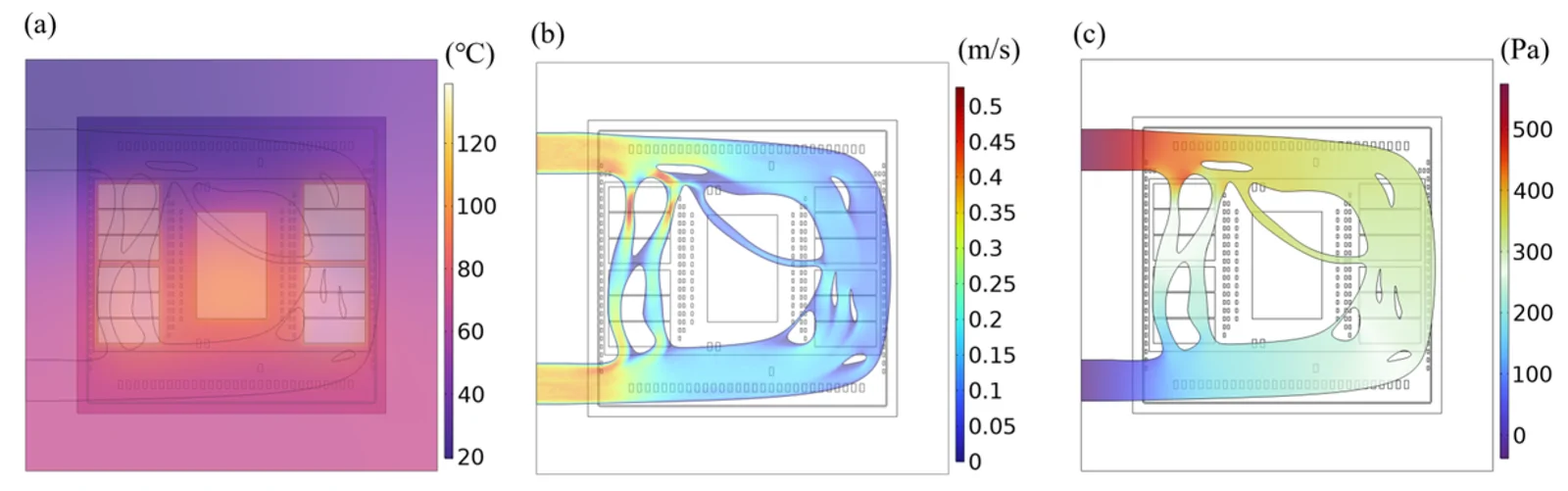
Temperature distribution of topology-optimized channel: (**a**) temperature distribution, (**b**) velocity distribution, (**c**) pressure distribution.

All six conventional channel configurations from Section 3.2 are applied to the non-uniform heat source, with simulation results shown in Figure 13. The topology-optimized channel simulation results are presented in Figure 14, with $\omega_{1}=0.4$, $\omega_{2}=0.3$, $\omega_{3}=0.3$, the high-temperature penalty exponent $P_{\mathrm{pen}}$ of 1.2, and a high-temperature penalty threshold of 130 °C. It should be noted that due to the concentrated nature of the heat source and the resulting high local heat flux densities, directly applying the high-temperature penalty threshold (75 °C) and high-temperature penalty exponent (1.9) from Section 3.2 would cause the high-temperature penalty objective term to dominate the other two objectives by a substantial margin, potentially leading to numerical divergence.

**Figure 15:**
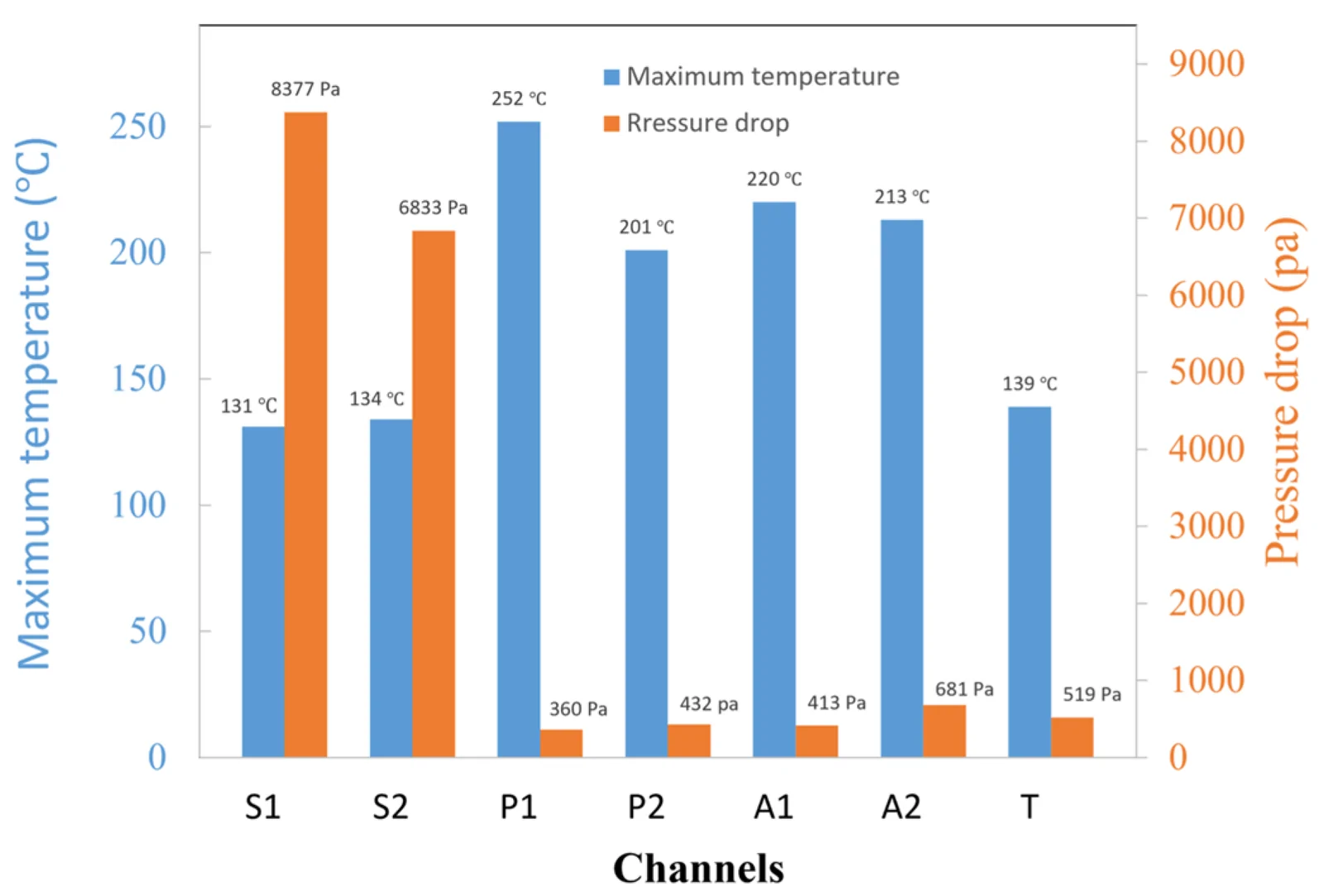
Maximum heat source temperature and pressure drop for different channel configurations under non-uniform heating.

The maximum heat source temperature and pressure drop for each channel are summarized in Figure 15. Among the conventional designs, serpentine channels continue to yield the lowest peak temperatures, at 131 °C and 134 °C, respectively. However, compared to the 73 °C achieved under uniform heating, this value is nearly 60 °C higher due to the concentrated heat sources. The corresponding pressure drops for the serpentine channels are 8377 Pa and 6833 Pa, maintaining their characteristically high hydraulic impedance. The numerical differences from the uniform heat source case arise in part from the temperature dependence of fluid viscosity, which introduces some nonlinearity.

The conventional channel geometries are identical to those in the previous section, and their temperature distributions are shown in Figure 13. Parallel and array channels perform even worse under non-uniform heating, with peak temperatures exceeding 200 °C. The root cause of such extreme temperatures is the highly concentrated heat sources—the distal CCDs receive almost no effective cooling and rapidly undergo thermal runaway. Although these configurations offer the lowest pressure drops at only 360 Pa, their thermal performance is entirely inadequate for practical applications involving high-power-density MCM processors.

In contrast, the topology-optimized channel demonstrates exceptional adaptability to the non-uniform thermal map. The optimized channel network is densely distributed over the CCDs, with pronounced branching enhancements in the CCD array region. Most critically, the channel widths above the CCDs near the inlet are noticeably narrower than those above the distal CCDs. This phenomenon reflects a sophisticated flow regulation strategy driven by the high-temperature penalty term: the optimizer deliberately reduces the cross-sectional area of the inlet-proximal channels to artificially increase their local hydraulic resistance, thereby “squeezing” more coolant toward the distal CCDs. Since the distal CCDs are farther from both inlet and outlet, their natural flow accessibility is poorer; this channel width modulation compensates for the inherent hydraulic imbalance, ensuring that all CCDs receive sufficient cooling flow and thereby maintaining the peak temperature within a controllable range. Ultimately, the topology-optimized channel achieves a maximum temperature of only 139 °C with a pressure drop of 519 Pa—both falling within desirable ranges.

This behavior is mechanistically consistent with the distal channel widening observed under uniform heating. However, under discrete CCD heat sources, the spatial enforcement effect is substantially stronger because the high-temperature penalty is highly sensitive to extreme temperature rises in any individual CCD. Consequently, the optimized topology autonomously evolves a flow distribution pattern inversely proportional to the local heat flux density and accessibility. This result profoundly reveals the indispensable role of the high-temperature penalty term in topology optimization for electronics cooling—it effectively embeds a principle of “thermal fairness,” compelling the optimizer to prevent any single hot spot from dominating the overall temperature response. This also implies that, unlike manually designed conventional channels, topology-optimized channels are not only hydraulically efficient but also inherently endowed with “heat-source awareness”—capable of automatically adapting to arbitrary power distributions without requiring heuristic adjustments or manual redesign.

## 4. Conclusions

This paper establishes a multi-objective topology optimization framework with a high-temperature penalty term for microchannel heat sink design. Parametric studies and comparative analyses under uniform and non-uniform heat sources yield the following conclusions:

(1) The high-temperature penalty term effectively suppresses localized extreme temperatures. Under uniform heating, the peak temperature drops from 117 °C (without penalty) to approximately 101 °C with a modest high-temperature penalty exponent ($P_{\mathrm{pen}}=1.1$). As the exponent increases, the peak temperature asymptotically approaches the threshold, while channels visibly migrate toward high-temperature regions, confirming the penalty’s targeted hot-spot suppression.

(2) Topology-optimized channels exhibit streamlined branching networks that balance thermal and hydraulic performance. Under uniform heating, the optimized channel achieves a maximum temperature of 79 °C and a pressure drop of 521 Pa—outperforming parallel and array channels thermally (peak temperatures > 129 °C) while maintaining far lower flow resistance than serpentine designs (pressure drops > 6000 Pa).

(3) For the highly concentrated, discrete heat sources of the AMD EPYC 9965 MCM Chiplet CPU, the topology-optimized channel demonstrates exceptional adaptability. It narrows inlet-proximal channels to redirect coolant toward distal CCDs, achieving a peak temperature of 139 °C and a pressure drop of 519 Pa. In contrast, conventional parallel and array channels exceed 200 °C due to inadequate distal cooling, rendering them unsuitable for high-power-density MCM processors.

(4) This framework provides an adaptive design pathway for liquid cold plates, effectively bridging high-performance thermal dissipation and engineering manufacturability. It should be noted that all numerical comparisons in this study are conducted under a consistent CFD solution environment, focusing on the relative gains among topological configurations and the validation of the design methodology. Nevertheless, we acknowledge that the current work is entirely based on numerical simulations and lacks independent experimental verification. To address this limitation, our future research will be directed towards constructing a dedicated experimental test rig, which will allow us to validate the optimized topologies under realistic operating conditions and further substantiate the practical value of the proposed approach.

## Figures and Tables

**Table 1:** Boundary and source conditions.

Inlet Flow Rate	Inlet/Outlet Dimensions	Inlet Coolant Temperature	Ambient Temperature	Convective Heat Transfer Coefficient (Air)	Uniform Heat Source Heat Dissipation	Non-Uniform Heat Source Heat Dissipation	
						I/O Die	CCD
0.15 L/min	1 × 10 mm	25 °C	25 °C	3 W(m^2^·K)	500 W	80 W	420 W

**Table 2:** Topology optimization computational parameters.

Da	q	L	β	$\theta_{\beta }$	Upper Solid Fraction
0.0001 [22]	0.01 [22]	10 mm	1.5	0.5	0.45

**Table 3:** Material parameters of liquid-cooled heat sink at 25 °C.

Component		Density (kg/m^3^)	Heat Conductivity (W(m·K))	Specific Heat (J/(kg·K))	Dynamic Viscosity (N·s/m^2^)
Cooling plate material		2700	230	340	—
Coolant		1000	0.59	4180	0.001
Uniform heat source	Heat source material	2700	230	340	—
Non-uniform heat source	Metal casing	2900	210	350	—
	Thermal interface layer	2600	3	975	—
	I/O Die	2330	150	700	—
	CCD	2330	150	700	—
	Substrate	1800	10	1100	—

**Table 4:** Geometric dimensions of each component.

Component	Length (mm)	Width (mm)	Height (mm)
Cooling plate	100	100	3
Topological domain	86	86	1
Inlet	10	7	1
Outlet	10	7	1
Uniform heat source	69.6	66.6	3
Metal casing	69.6	66.6	0.5
Thermal interface layer	67	42	0.5
CCD	15	6	1
I/O Die	26	17	1
Substrate	75	72	1

**Table 5:** Mesh independence.

Total mesh elements (million)	3.9	4.6	5.0	7.2	9.6	14.5
Maximum temperature (°C)	78.8 °C	78.9 °C	78.9 °C	78.9 °C	78.9 °C	78.9 °C
Temperature bias	0.1%	0.1%	<0.1%	<0.1%	<0.1%	<0.1%
Maximum pressure drop (Pa)	515 Pa	518 Pa	520 Pa	521 Pa	521 Pa	521 Pa
Pressure drop bias	1.2%	0.6%	0.2%	<0.1%	<0.1%	<0.1%

## Data Availability

The original contributions presented in this study are included in the article. Further inquiries can be directed to the corresponding author.
